# Supplementary material for: A practical evaluation of statistical methods for the analysis of patient reported outcomes in an observational pharmaceutical study
Source: PLoS One. 2026 Mar 18;21(3):e0344968. doi: 10.1371/journal.pone.0344968 (PMC12998841; doi:10.1371/journal.pone.0344968)
Supplement: S1 Methods — (DOCX) [file pone.0344968.s001.docx]

***Supplementary Methods***

Unweighted generalised estimating equation analysis

To compare the results of alternative GEE extensions to MAR missingness, we performed an unweighted GEE analysis on a dataset with missing MCS and PCS values imputed with multiple imputation. We used the “amelia” R package to impute missing values, using the following variables: time from treatment initiation, age, sex, HIV viral load at treatment initiation, number of neuropsychiatric comorbidities, number of physical comorbidities, presentation with advanced HIV, race and other MCS and PCS scores. We then performed the best fitting weighted GEE models with a continuous time variable (fractional polynomial time variable for MCS and polynomial time variable for PCS), without the weights. We compared the results to the respective wGEE models.

*MNAR sensitivity analysis*

To check if the conclusions of the LMMs and wGEEs were robust to missing data being MNAR, multiple sensitivity analyses were performed, recreating alternative possible MNAR scenarios.

Following the process outlined in Salazar *et al.* (2016) [1] and using the “amelia” R package as outlined above, we imputed values for missing MCS and PCS observations. As they recommend for MNAR sensitivity analysis of the SF-36, a bias given by a normal distribution with a mean of 5 (sd = 1) was added or subtracted from the imputed responses. In each sensitivity analysis, this was performed either on the imputed observations that were intermittent missingness, dropout missingness, or to both intermittent and dropout missingness. The wGEE and LMM models were re-run with the modified datasets, and the quantitative results and qualitative conclusions were compared.

***References***

1. Salazar A, Ojeda B, Dueñas M, Fernández F, Failde I. Simple generalized estimating equations (GEEs) and weighted generalized estimating equations (WGEEs) in longitudinal studies with dropouts: guidelines and implementation in R. Stat Med. 2016
