# Supplementary material for: A practical evaluation of statistical methods for the analysis of patient reported outcomes in an observational pharmaceutical study
Source: PLoS One. 2026 Mar 18;21(3):e0344968. doi: 10.1371/journal.pone.0344968 (PMC12998841; doi:10.1371/journal.pone.0344968)

***Analytical Processes***

***Figure S1A.*** ***Visual representation of the analytical process for the Paired Difference Test.*** *Grey boxes represent the actions taken. M = month of follow up.*


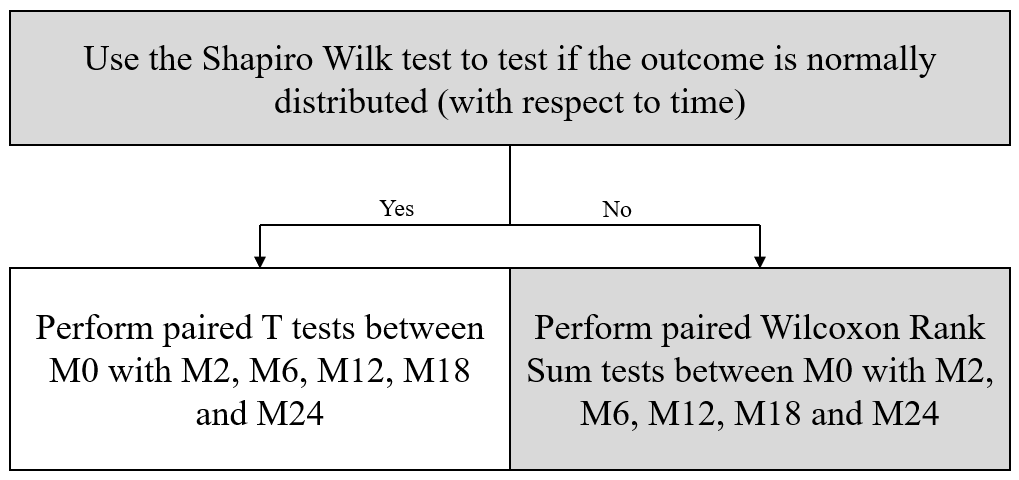


***Figure S1B.*** ***Visual representation of the analytical process for the Repeated Measures ANOVA***. *Grey boxes represent the actions taken.*


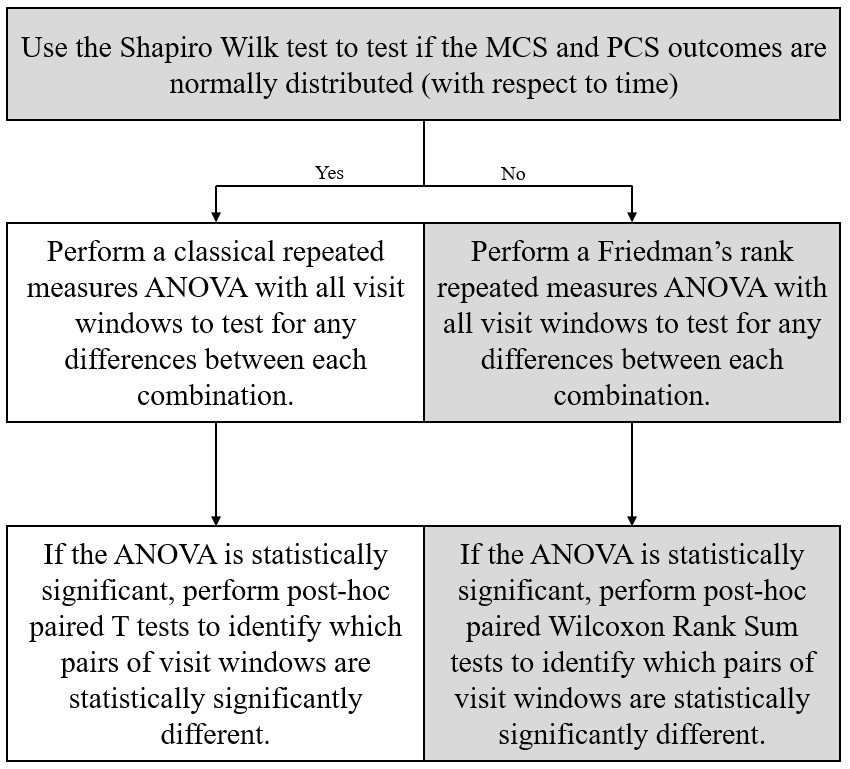


***Figure S1C.*** ***Visual representation of the analytical process for the Linear Mixed Model***. *Grey boxes represent the actions taken.*


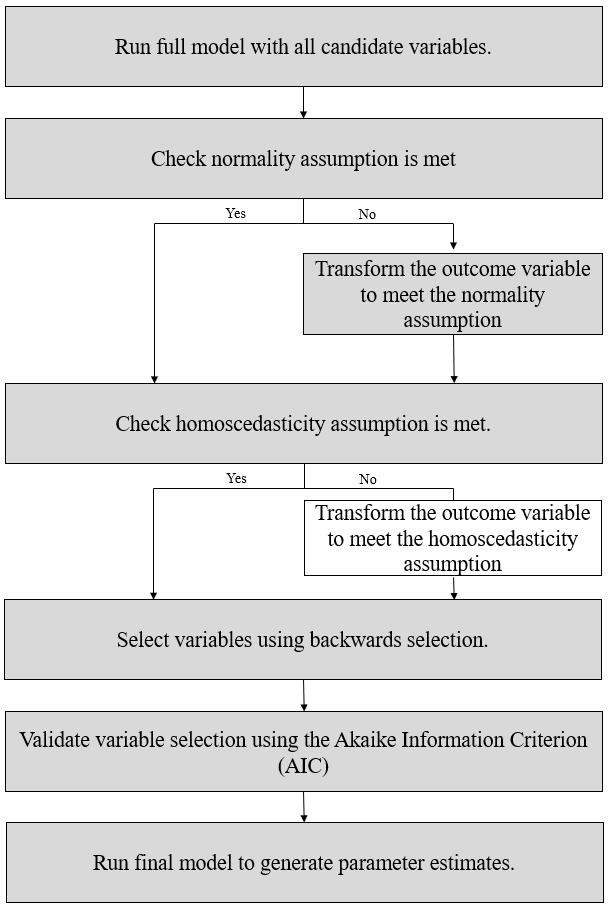


***Figure S1D.*** ***Visual representation of the analytical process for the Generalised Estimating Equation (categorical time variable)***. *Grey boxes represent the actions taken.*


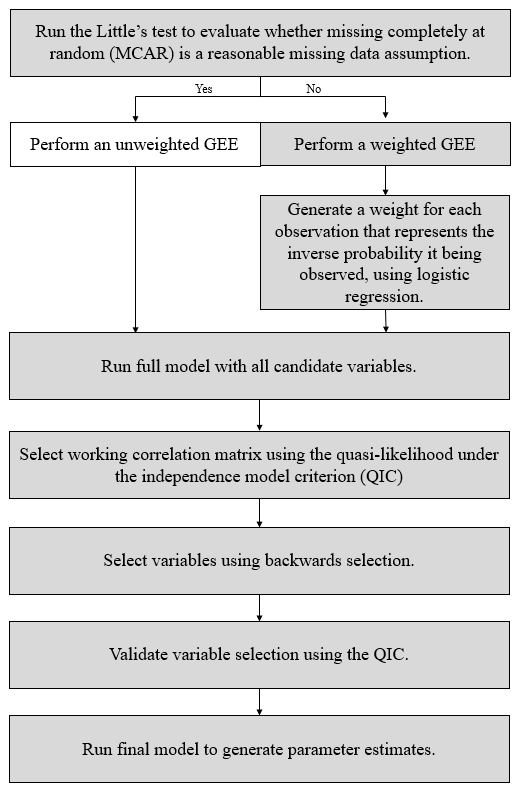


***Figure S1E.*** ***Visual representation of the analytical process for the Generalised Estimating Equation (continuous time variable)***. *Grey boxes represent the actions taken.*


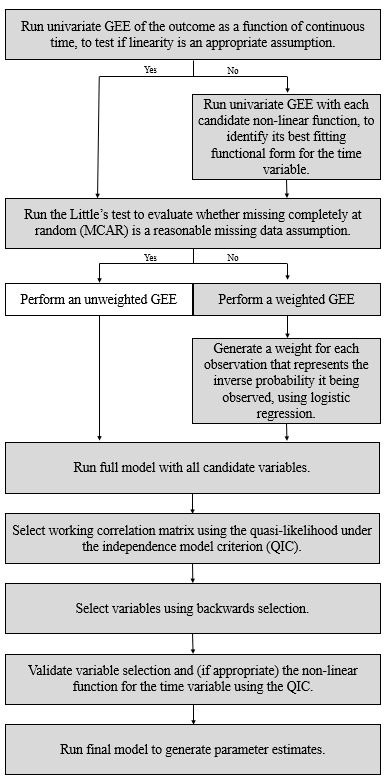

Supplement: S1 Fig — (DOCX) [file pone.0344968.s002.docx]
