# Supplementary material for: A practical evaluation of statistical methods for the analysis of patient reported outcomes in an observational pharmaceutical study
Source: PLoS One. 2026 Mar 18;21(3):e0344968. doi: 10.1371/journal.pone.0344968 (PMC12998841; doi:10.1371/journal.pone.0344968)

***Component Score Distributions***

***Figure S2A.*** ***Mental Component Score distribution over follow-up.***


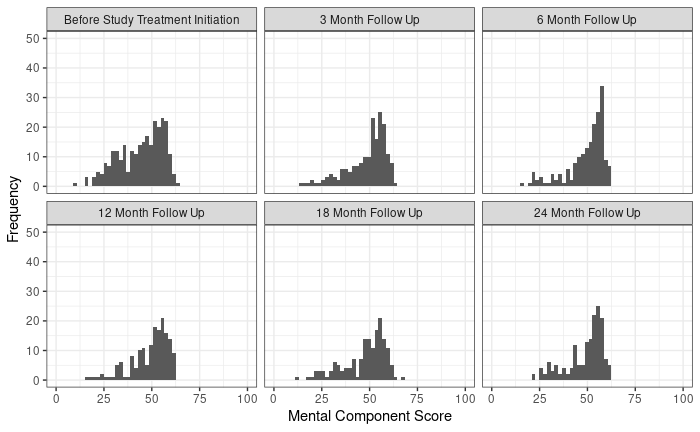


***Figure S2B.*** ***Physical Component Score distribution over follow-up.***


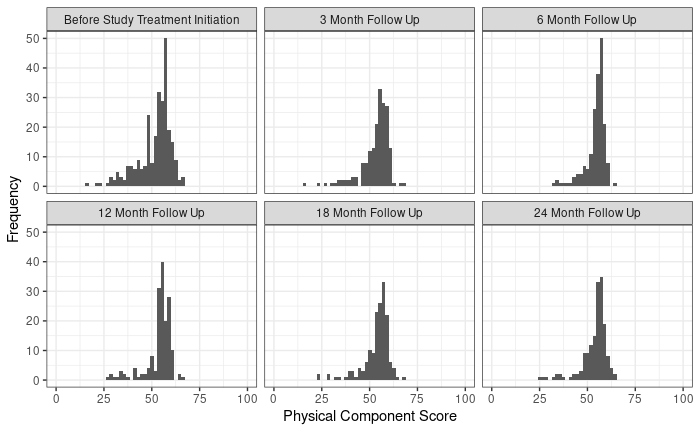

Supplement: S2 Fig — (DOCX) [file pone.0344968.s003.docx]
