# Supplementary material for: A practical evaluation of statistical methods for the analysis of patient reported outcomes in an observational pharmaceutical study
Source: PLoS One. 2026 Mar 18;21(3):e0344968. doi: 10.1371/journal.pone.0344968 (PMC12998841; doi:10.1371/journal.pone.0344968)

***Balanced and Unbalanced MCS and PCS Across Visits***

***Figure S3. Median Mental Component Score (MCS) and Physical Component Score (PCS) at each of the six visit windows in the population providing balanced SF-36 data, and the population not providing balanced SF-36 data.*** *The balanced population is analysed in the Friedman’s ANOVA, while the unbalanced population is not.*


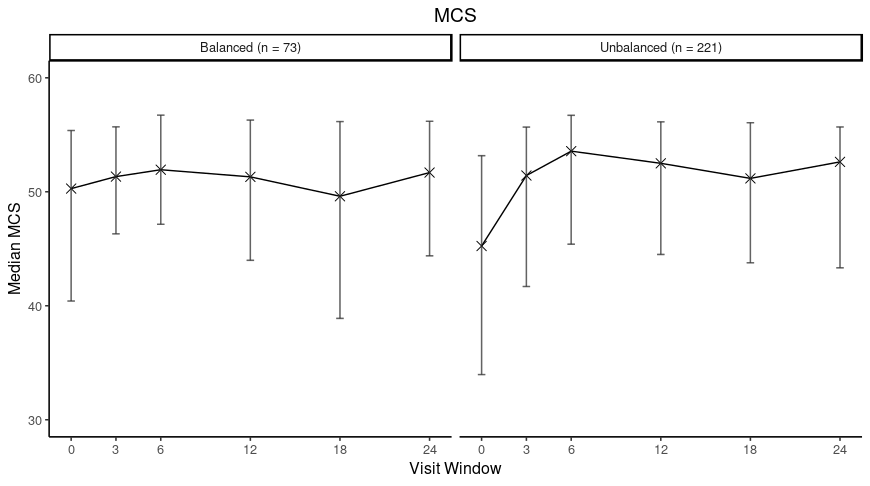

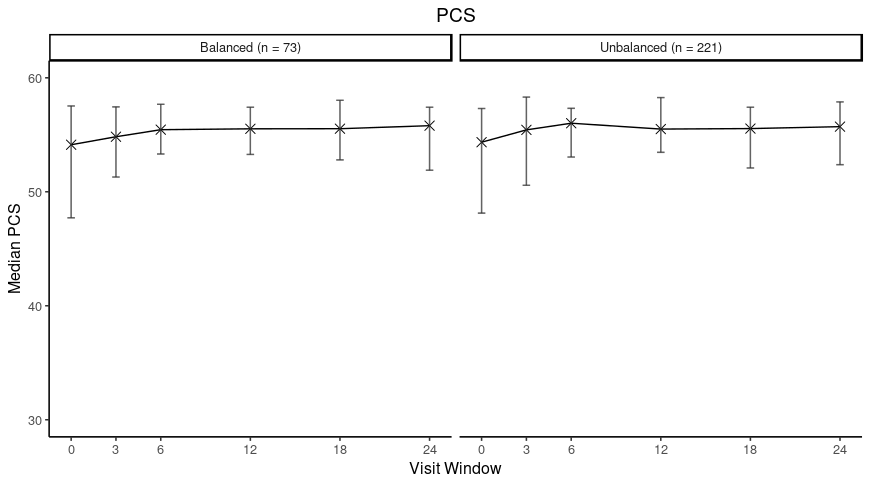

Supplement: S3 Fig — (DOCX) [file pone.0344968.s004.docx]
