# Supplementary material for: A practical evaluation of statistical methods for the analysis of patient reported outcomes in an observational pharmaceutical study
Source: PLoS One. 2026 Mar 18;21(3):e0344968. doi: 10.1371/journal.pone.0344968 (PMC12998841; doi:10.1371/journal.pone.0344968)
Supplement: S4 Fig — (DOCX) [file pone.0344968.s005.docx]

***Model Diagnostics***

***Figure S4A. Model diagnostic plots for the Mental Component Score (MCS) categorical-time linear mixed model.*** *A) Residuals vs observations, B) Residuals vs fitted values, C) Q-Q plot of residuals, D) Q-Q plot of random effects.*


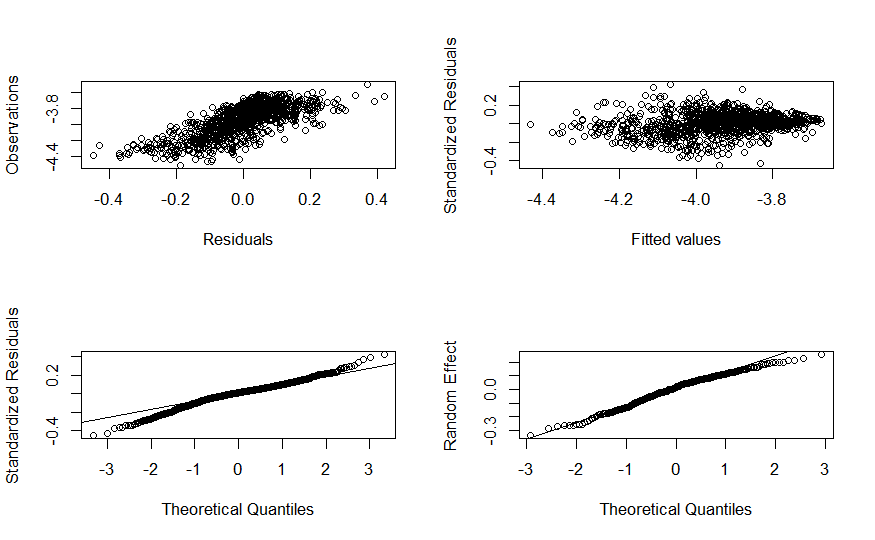


**A**

**D**

**C**

**B**

***Figure S4B. Model diagnostic plots for the Physical Component Score (PCS) categorical-time linear mixed model.*** *A) Residuals vs observations, B) Residuals vs fitted values, C) Q-Q plot of residuals, D) Q-Q plot of random effects.*


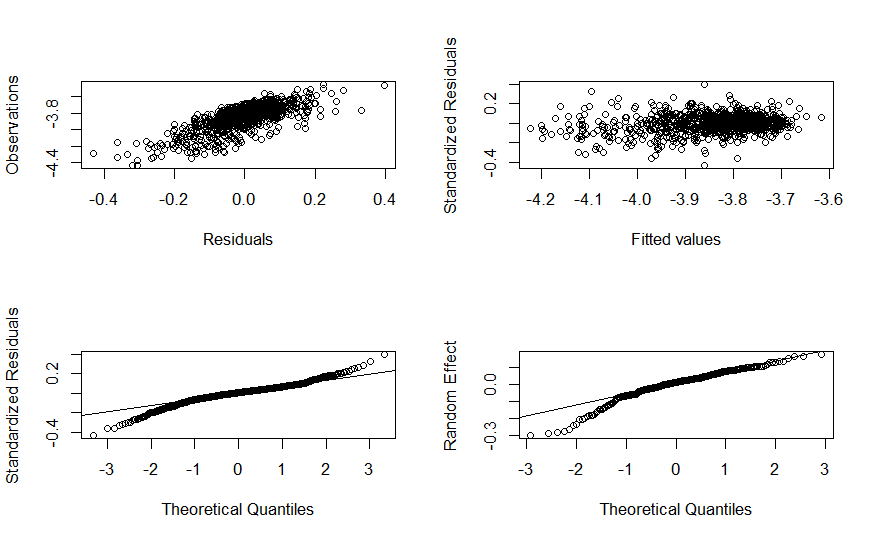


**A**

**D**

**C**

**B**

***Figure S4C. Model diagnostic plots for the Mental Component Score (MCS) continuous-time linear mixed model.*** *A) Residuals vs observations, B) Residuals vs fitted values, C) Q-Q plot of residuals, D) Q-Q plot of random effects.*


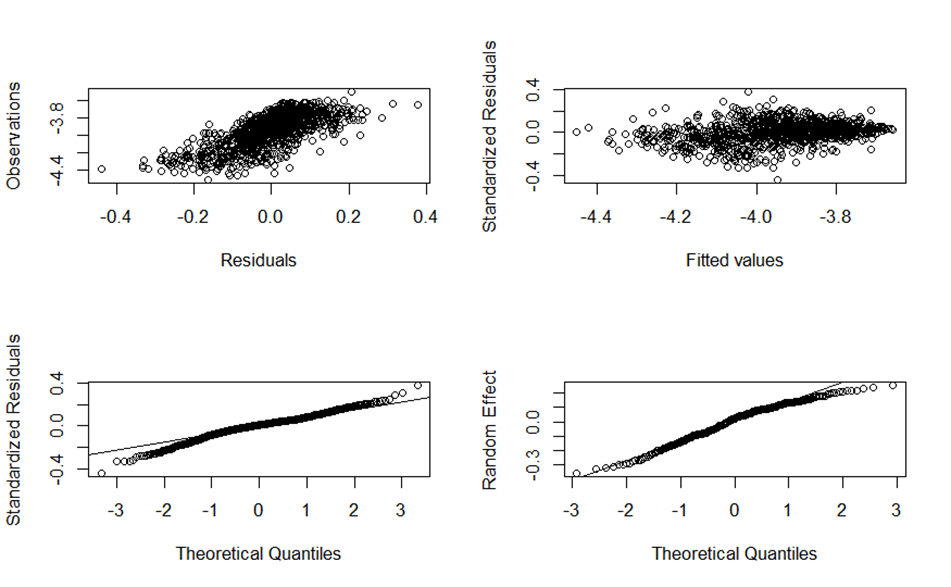


**A**

**D**

**C**

**B**

***Figure S4D. Model diagnostic plots for the Physical Component Score (PCS) continuous-time linear mixed model.*** *A) Residuals vs observations, B) Residuals vs fitted values, C) Q-Q plot of residuals, D) Q-Q plot of random effects.*


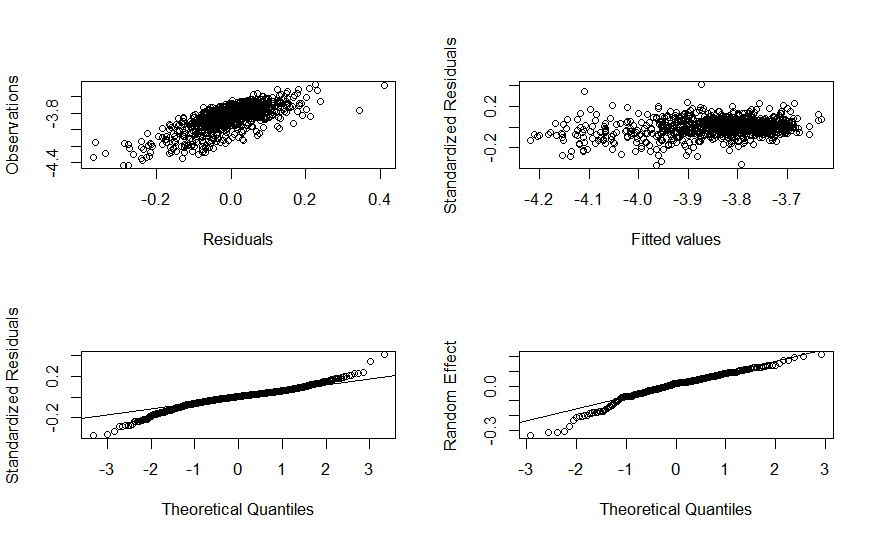


**A**

**D**

**C**

**B**
