# Supplementary material for: A practical evaluation of statistical methods for the analysis of patient reported outcomes in an observational pharmaceutical study
Source: PLoS One. 2026 Mar 18;21(3):e0344968. doi: 10.1371/journal.pone.0344968 (PMC12998841; doi:10.1371/journal.pone.0344968)
Supplement: S1 Table — (DOCX) [file pone.0344968.s006.docx]

***Mental Component LMM and wGEE Regression Estimates***

**Table S1.** ***Regression estimates for the mental component score (MCS) linear mixed model (LMM) and weighted generalised estimating equation (wGEE)^1^.***

|  | LMM (MCSt) | | | | wGEE (MCS) | | | |
| --- | --- | --- | --- | --- | --- | --- | --- | --- |
|  | Estimate | SE | T value | P value | Estimate | SE | Wald | P value |
| (Intercept) | -3.98 | 0.01 | -325.52 | <0.001 | 45.39 | 0.69 | 4315.53 | <0.001 |
| Time (3 Months) | 0.07 | 0.01 | 5.69 | <0.001 | 3.97 | 0.66 | 36.51 | <0.001 |
| Time (6 Months) | 0.08 | 0.01 | 6.49 | <0.001 | 4.44 | 0.71 | 39.57 | <0.001 |
| Time (12 Months) | 0.08 | 0.01 | 6.28 | <0.001 | 4.92 | 0.76 | 42.18 | <0.001 |
| Time (18 Months) | 0.06 | 0.01 | 4.00 | <0.001 | 3.26 | 0.85 | 14.63 | <0.001 |
| Time (24 Months) | 0.07 | 0.02 | 4.69 | <0.001 | 4.98 | 0.82 | 37.08 | <0.001 |
| Sex (Female) | 4.52E-04 | 0.04 | 0.01 | 0.991 | 0.38 | 1.91 | 0.04 | 0.841 |
| Age (decades) | 0.01 | 0.01 | 1.36 | 0.174 | 0.38 | 0.44 | 0.76 | 0.384 |
| Number of Mental Comorbidities | -0.13 | 0.02 | -5.93 | <0.001 | -6.90 | 1.37 | 25.27 | <0.001 |
| Log(HIV RNA) | -0.02 | 0.01 | -3.17 | 0.002 | -0.89 | 0.29 | 9.66 | 0.002 |
| x Time (3 Months) | 0.02 | 0.01 | 3.17 | 0.002 | 0.81 | 0.29 | 7.74 | 0.005 |
| x Time (6 Months) | 0.02 | 0.01 | 3.39 | 0.001 | 0.81 | 0.29 | 7.85 | 0.005 |
| x Time (12 Months) | 0.01 | 0.01 | 2.20 | 0.028 | 0.73 | 0.34 | 4.59 | 0.032 |
| x Time (18 Months) | 3.54E-03 | 0.01 | 0.61 | 0.542 | 0.35 | 0.34 | 1.03 | 0.310 |
| x Time (24 Months) | 0.01 | 0.01 | 1.22 | 0.224 | 0.51 | 0.35 | 2.14 | 0.143 |

*^1^The LMM models the transformed MCS (MCSt), while the wGEE models the untransformed MCS.*
