# Supplementary material for: A practical evaluation of statistical methods for the analysis of patient reported outcomes in an observational pharmaceutical study
Source: PLoS One. 2026 Mar 18;21(3):e0344968. doi: 10.1371/journal.pone.0344968 (PMC12998841; doi:10.1371/journal.pone.0344968)
Supplement: S2 Table — (DOCX) [file pone.0344968.s007.docx]

***Physical Component LMM and wGEE Regression Estimates***

***Table S2. Regression estimates for the physical component score (PCS) linear mixed model (LMM) and weighted generalised estimating equation (wGEE)^1^.*** *The LMM models the transformed PCS (PCSt), while the wGEE models the untransformed PCS.*

|  | LMM (PCSt) | | | | wGEE (PCS) | | | |
| --- | --- | --- | --- | --- | --- | --- | --- | --- |
|  | Estimate | SE | T value | P value | Estimate | SE | Wald | P value |
| (Intercept) | -3.71 | 0.02 | -154.04 | <0.001 | 59.28 | 1.28 | 2137.35 | <0.001 |
| Time (3 Months) | 0.02 | 0.01 | 2.48 | 0.014 | 1.25 | 0.50 | 6.23 | 0.013 |
| Time (6 Months) | 0.03 | 0.01 | 3.22 | 0.001 | 1.76 | 0.51 | 12.16 | <0.001 |
| Time (12 Months) | 0.02 | 0.01 | 2.10 | 0.036 | 1.45 | 0.54 | 7.12 | 0.008 |
| Time (18 Months) | 0.03 | 0.01 | 2.35 | 0.019 | 1.73 | 0.56 | 9.51 | 0.002 |
| Time (24 Months) | 0.02 | 0.01 | 2.03 | 0.042 | 1.70 | 0.60 | 7.93 | 0.005 |
| Sex (Female) | -0.05 | 0.03 | -1.81 | 0.071 | -1.70 | 1.61 | 1.10 | 0.293 |
| Age (decades) | -0.03 | 0.01 | -5.26 | <0.001 | -1.56 | 0.33 | 22.03 | <0.001 |
| Number of Physical Comorbidities | -0.01 | 0.01 | -2.87 | 0.004 | -0.78 | 0.31 | 6.50 | 0.011 |
| Advanced HIV | -0.09 | 0.02 | -4.15 | <0.001 | -5.28 | 1.61 | 10.75 | 0.001 |
| x Time (3 Months) | 0.06 | 0.02 | 2.53 | 0.012 | 3.84 | 1.87 | 4.20 | 0.040 |
| x Time (6 Months) | 0.10 | 0.02 | 4.31 | <0.001 | 5.78 | 1.33 | 18.92 | <0.001 |
| x Time (12 Months) | 0.11 | 0.03 | 4.29 | <0.001 | 6.33 | 1.89 | 11.23 | 0.001 |
| x Time (18 Months) | 0.09 | 0.03 | 3.39 | 0.001 | 4.80 | 1.76 | 7.48 | 0.006 |
| x Time (24 Months) | 0.08 | 0.03 | 2.70 | 0.007 | 4.13 | 1.68 | 6.07 | 0.014 |
| Log(HIV RNA) | -0.01 | 3.58E-03 | -2.80 | 0.005 | -0.54 | 0.22 | 6.10 | 0.014 |
| x Time (3 Months) | 0.01 | 3.85E-03 | 3.35 | 0.001 | 0.69 | 0.24 | 8.39 | 0.004 |
| x Time (6 Months) | 0.01 | 3.86E-03 | 2.49 | 0.013 | 0.50 | 0.19 | 6.91 | 0.009 |
| x Time (12 Months) | 0.01 | 4.20E-03 | 3.03 | 0.003 | 0.73 | 0.24 | 9.36 | 0.002 |
| x Time (18 Months) | 0.01 | 4.36E-03 | 2.41 | 0.016 | 0.59 | 0.22 | 6.89 | 0.009 |
| x Time (24 Months) | 0.02 | 4.67E-03 | 3.84 | <0.001 | 0.93 | 0.24 | 14.98 | <0.001 |

*^1^The LMM models the transformed MCS (MCSt), while the wGEE models the untransformed MCS.*
