# Supplementary material for: A practical evaluation of statistical methods for the analysis of patient reported outcomes in an observational pharmaceutical study
Source: PLoS One. 2026 Mar 18;21(3):e0344968. doi: 10.1371/journal.pone.0344968 (PMC12998841; doi:10.1371/journal.pone.0344968)
Supplement: S3 Table — (DOCX) [file pone.0344968.s008.docx]

***Probability of SF-36 Observation Logistic Regression Results***

***Table S3A. Model results for baseline (Month 0) logistic regression model for the probability that an individual would have an SF-36 observation.*** *The inverse of the predicted probabilities was used to generate the weights for the wGEE. SE = standard error; LCI = lower confidence interval; UCI = upper confidence interval. Hosmer and Lemeshow goodness of fit (GOF) test p>0.05.*

|  | Estimate | SE | Wald | P value | LCI | UCI |
| --- | --- | --- | --- | --- | --- | --- |
| (Intercept) | 8.99E-12 | 5448.18 | -4.67E-03 | 9.96E-01 | NA | 3.76E+197 |
| Log(HIV RNA) | 1.49 | 0.16 | 2.42 | 1.54E-02 | 1.08 | 2.09 |
| Sex (Male) | 3.80E+07 | 5448.18 | 3.20E-03 | 9.97E-01 | 1.78E-215 | NA |
| Age (years) | 1.02 | 0.04 | 0.44 | 6.60E-01 | 9.36E-01 | 1.09 |
| Number of Neuropsychiatric Comorbidities | 3.58E-08 | 3255.50 | -5.27E-03 | 9.96E-01 | NA | 2.53E+125 |
| Number of Physical Comorbidities | 0.26 | 1.01 | -1.33 | 0.18 | 1.43E-02 | 1.08 |
| Advanced HIV (yes) | 1.23E-08 | 3394.44 | -5.37E-03 | 9.96E-01 | NA | 4.07E+130 |

***Table S3B. Model results for Month 3 logistic regression model for the probability that an individual would have an SF-36 observation.*** *The inverse of the predicted probabilities was used to generate the weights for the wGEE. SE = standard error; LCI = lower confidence interval; UCI = upper confidence interval. Hosmer and Lemeshow goodness of fit (GOF) test p>0.05.*

|  | Estimate | SE | Wald | P value | LCI | UCI |
| --- | --- | --- | --- | --- | --- | --- |
| (Intercept) | 4.55 | 2.31 | 0.66 | 5.12E-01 | 0.05 | 426.91 |
| Log(HIV RNA) | 1.08 | 0.09 | 0.83 | 4.06E-01 | 0.91 | 1.29 |
| Sex (Male) | 0.07 | 0.67 | -3.91 | 9.37E-05 | 0.02 | 0.27 |
| Age (years) | 0.98 | 0.02 | -1.12 | 2.63E-01 | 0.94 | 1.02 |
| Number of Neuropsychiatric Comorbidities | 0.52 | 0.73 | -0.91 | 3.65E-01 | 0.1 | 1.84 |
| Number of Physical Comorbidities | 0.48 | 0.3 | -2.48 | 1.31E-02 | 0.25 | 0.81 |
| Month 0 MCS | 1.00 | 0.03 | -0.01 | 9.90E-01 | 0.953 | 1.05 |
| Month 0 PCS | 0.98 | 0.02 | -0.87 | 3.83E-01 | 0.95 | 1.02 |
| Advanced HIV (yes) | 1.53 | 0.51 | 0.85 | 3.97E-01 | 0.54 | 4.02 |

***Table S3C. Model results for Month 6 logistic regression model for the probability that an individual would have an SF-36 observation.*** *The inverse of the predicted probabilities was used to generate the weights for the wGEE. SE = standard error; LCI = lower confidence interval; UCI = upper confidence interval. Hosmer and Lemeshow goodness of fit (GOF) test p>0.05.*

|  | Estimate | SE | Wald | P value | LCI | UCI |
| --- | --- | --- | --- | --- | --- | --- |
| (Intercept) | 209.09 | 1.99 | 2.69 | 0.01 | 4.43 | 1.11E+04 |
| Log(HIV RNA) | 1.08 | 0.07 | 1.09 | 0.28 | 0.94 | 1.25 |
| Sex (Male) | 0.14 | 0.62 | -3.19 | 1.44E-03 | 0.04 | 0.47 |
| Age (years) | 0.96 | 0.02 | -2.18 | 0.03 | 0.93 | 9.95E-01 |
| Number of Neuropsychiatric Comorbidities | 0.96 | 0.02 | -3.00 | 2.67E-03 | 0.93 | 0.98 |
| Number of Physical Comorbidities | 0.96 | 0.02 | -1.74 | 0.08 | 0.92 | 1.01 |
| Most recent MCS | 0.72 | 0.53 | -0.61 | 0.54 | 0.23 | 1.92 |
| Most recent PCS | 0.5 | 0.23 | -2.97 | 2.97E-03 | 0.31 | 0.76 |
| Advanced HIV (yes) | 1.84 | 0.43 | 1.42 | 0.15 | 0.78 | 4.23 |

***Table S3D. Model results for Month 12 logistic regression model for the probability that an individual would have an SF-36 observation.*** *The inverse of the predicted probabilities was used to generate the weights for the wGEE. SE = standard error; LCI = lower confidence interval; UCI = upper confidence interval. Hosmer and Lemeshow goodness of fit (GOF) test p>0.05.*

|  | Estimate | SE | Wald | P value | LCI | UCI |
| --- | --- | --- | --- | --- | --- | --- |
| (Intercept) | 94.17 | 1.39 | 3.28 | 1.05E-03 | 6.61 | 1555.59 |
| Log(HIV RNA) | 9.97E-01 | 0.06 | -0.05 | 0.96 | 0.88 | 1.13 |
| Sex (Male) | 0.31 | 0.5 | -2.12 | 0.03 | 0.1 | 0.94 |
| Age (years) | 0.97 | 0.015 | -1.91 | 0.06 | 0.94 | 1 |
| Number of Neuropsychiatric Comorbidities | 0.6 | 0.47 | -1.03 | 0.3 | 0.23 | 1.46 |
| Number of Physical Comorbidities | 0.54 | 0.19 | -3.28 | 1.02E-03 | 0.36 | 0.76 |
| Most recent MCS | 0.98 | 0.02 | -1.07 | 0.28 | 0.95 | 1.02 |
| Most recent PCS | 0.96 | 0 | -2.21 | 0.03 | 0.92 | 0.99 |
| Advanced HIV (yes) | 1.6 | 0.38 | 1.24 | 0.22 | 0.75 | 3.35 |

***Table S3E. Model results for Month 18 logistic regression model for the probability that an individual would have an SF-36 observation.*** *The inverse of the predicted probabilities was used to generate the weights for the wGEE. SE = standard error; LCI = lower confidence interval; UCI = upper confidence interval. Hosmer and Lemeshow goodness of fit (GOF) test p>0.05.*

|  | Estimate | SE | Wald | P value | LCI | UCI |
| --- | --- | --- | --- | --- | --- | --- |
| (Intercept) | 462.52 | 1.41 | 4.35 | 1.34E-05 | 32.11 | 8235.73 |
| Log(HIV RNA) | 0.94 | 0.06 | -1.12 | 0.26 | 0.83 | 1.05 |
| Sex (Male) | 0.26 | 0.56 | -2.45 | 0.01 | 0.08 | 0.76 |
| Age (years) | 0.96 | 0.02 | -2.52 | 0.01 | 0.94 | 0.99 |
| Number of Neuropsychiatric Comorbidities | 2.01 | 0.41 | 1.72 | 0.09 | 0.9 | 4.47 |
| Number of Physical Comorbidities | 0.51 | 0.19 | -3.62 | 2.90E-04 | 0.34 | 0.72 |
| Most recent MCS | 1.03 | 0.02 | 1.26 | 0.21 | 0.99 | 1.07 |
| Most recent PCS | 0.91 | 0.02 | -4.29 | 1.80E-05 | 0.87 | 0.95 |
| Advanced HIV (yes) | 1.19 | 0.39 | 0.45 | 0.65 | 0.55 | 2.51 |

***Table S3F. Model results for Month 24 logistic regression model for the probability that an individual would have an SF-36 observation.*** *The inverse of the predicted probabilities was used to generate the weights for the wGEE. SE = standard error; LCI = lower confidence interval; UCI = upper confidence interval. Hosmer and Lemeshow goodness of fit (GOF) test p>0.05.*

|  | Estimate | SE | Wald | P value | LCI | UCI |
| --- | --- | --- | --- | --- | --- | --- |
| (Intercept) | 156.83 | 1.29 | 3.92 | 8.88E-05 | 13.67 | 2188.35 |
| Log(HIV RNA) | 0.95 | 0.054 | -0.99 | 0.32 | 0.85 | 1.05 |
| Sex (Male) | 0.39 | 0.54 | -1.73 | 0.08 | 0.13 | 1.1 |
| Age (years) | 0.97 | 0.01 | -1.17 | 0.24 | 0.96 | 1.01 |
| Number of Neuropsychiatric Comorbidities | 1.26 | 0.34 | 0.69 | 0.49 | 0.65 | 2.44 |
| Number of Physical Comorbidities | 0.7 | 0.12 | -2.34 | 0.02 | 0.59 | 0.95 |
| Most recent MCS | 1 | 0.02 | 1.58 | 0.11 | 1 | 1.06 |
| Most recent PCS | 0.9 | 0 | -4.68 | 2.84E-06 | 0.88 | 0.95 |
| Advanced HIV (yes) | 1.2 | 0.35 | 0.56 | 0.58 | 0.61 | 2.43 |
