# Supplementary material for: A practical evaluation of statistical methods for the analysis of patient reported outcomes in an observational pharmaceutical study
Source: PLoS One. 2026 Mar 18;21(3):e0344968. doi: 10.1371/journal.pone.0344968 (PMC12998841; doi:10.1371/journal.pone.0344968)
Supplement: S4 Table — (DOCX) [file pone.0344968.s009.docx]

***Final wGEE QIC***

***Table S4. Quasi-likelihood under the independence model criterion (QIC) values for the final wGEE models with all selected covariates.*** *The lowest value and therefore the selected structure for each model was the unstructured correlation matrix.*

|  | **QIC** | | | |
| --- | --- | --- | --- | --- |
| **Correlation structure** | **Categorical - MCS** | **Categorical - PCS** | **Continuous - MCS** | **Continuous - PCS** |
| **Independent** | 1218.951 | 1280.759 | 1198.898 | 1261.874 |
| **Exchangeable** | 1213.678 | 1271.179 | 1197.639 | 1253.573 |
| **Unstructured** | 1206.185 | 1269.321 | 1191.167 | 1252.348 |
| **Autoregressive 1** | 1222.707 | 1271.378 | 1207.759 | 1253.378 |
