# Supplementary material for: A practical evaluation of statistical methods for the analysis of patient reported outcomes in an observational pharmaceutical study
Source: PLoS One. 2026 Mar 18;21(3):e0344968. doi: 10.1371/journal.pone.0344968 (PMC12998841; doi:10.1371/journal.pone.0344968)
Supplement: S5 Table — (DOCX) [file pone.0344968.s010.docx]

***Sensitivity Analyses***

***Table S5A. Sensitivity analysis for the MCS weighted generalised estimating equation model with time modelled using a fractional polynomial.*** *1) Weighted GEE regression output, as presented in the main text; 2) Unweighted GEE regression output, with missing values imputed using multiple imputation; 3) Weighted GEE regression output with missing MCS values imputed using multiple imputation and a bias with a mean of 5 points added;* 4*) Weighted GEE regression output with missing MCS values imputed using multiple imputation and a bias with a mean of 5 points subtracted.*

|  | 1) wGEE | | 2) MI GEE | | 3) MI wGEE +5 | | 4) MI wGEE -5 | |
| --- | --- | --- | --- | --- | --- | --- | --- | --- |
|  | Est. | P-value | Est. | P-value | Est. | P-value | Est. | P-value |
| (Intercept) | 49.86 | <0.001 | 50.55 | <0.001 | 52.31 | <0.001 | 48.97 | <0.001 |
| (Time + 0.1)/10)^-2^ | -4.27e-4 | <0.001 | 4.86e-4 | <0.001 | 5.90e-4 | <0.001 | 3.51e-4 | <0.001 |
| Sex (Female) | 0.41 | 0.832 | 0.77 | 0.679 | 0.39 | 0.779 | 0.25 | 0.900 |
| Age (decades) | 0.37 | 0.399 | 0.54 | 0.167 | 0.55 | 0.211 | 0.65 | 0.100 |
| Number of Neuropsychiatric Comorbidities | -6.87 | <0.001 | -7.68 | <0.001 | -8.66 | <0.001 | -7.58 | <0.001 |
| Log(HIV RNA) | -0.28 | 0.254 | -0.30 | 0.187 | -0.13 | 0.720 | -0.34 | 0.067 |
| x (Time + 0.1)/10)^-2^ | -7.54e-5 | 0.006 | 5.58e-5 | 0.059 | -6.72e-5 | 0.053 | -4.85e-5 | 0.072 |

***Table S5B. Sensitivity analysis for the PCS weighted generalised estimating equation model with time modelled using a fractional polynomial.*** *1) Weighted GEE regression output, as presented in the main text; 2) Unweighted GEE regression output, with missing values imputed using multiple imputation; 3) Weighted GEE regression output with missing PCS values imputed using multiple imputation and a bias with a mean of 5 points added;* 4*) Weighted GEE regression output with missing PCS values imputed using multiple imputation and a bias with a mean of 5 points subtracted.*

|  | wGEE | | MI GEE | | MI wGEE +5 | | MI wGEE -5 | |
| --- | --- | --- | --- | --- | --- | --- | --- | --- |
|  | Est. | P-value | Est. | P-value | Est. | P-value | Est. | P-value |
| (Intercept) | 59.56 | <0.001 | 53.55 | <0.001 | 53.80 | <0.001 | 53.45 | <0.001 |
| Time (months) | 0.43 | 0.004 | 0.58 | 0.007 | 0.79 | <0.001 | 0.45 | 0.049 |
| Time (months)^2^ | -0.03 | 0.04 | -0.04 | 0.058 | -0.06 | 0.005 | -0.037 | 0.097 |
| Time (months)^3^ | 6.60e-4 | 0.111 | 9.70e-4 | 0.136 | 1.30e-3 | 0.024 | 7.01e-4 | 0.205 |
| Sex (Female) | -4.68 | 0.071 | -3.99 | 0.082 | -3.41 | 0.139 | -3.44 | 0.153 |
| x Time (months) | 1.55 | 0.093 | 0.31 | 0.750 | 0.43 | 0.646 | -0.31 | 0.780 |
| x Time (months)^2^ | -0.16 | 0.026 | -0.03 | 0.723 | -0.05 | 0.576 | 0.02 | 0.811 |
| x Time (months)^3^ | 4.19e-3 | 0.005 | 9.97e-4 | 0.675 | 1.53e-3 | 0.507 | 2.41e-4 | 0.929 |
| Age (decades) | -1.59 | <0.001 | -1.61 | <0.001 | -1.61 | <0.001 | -1.64 | <0.001 |
| Number of Physical Comorbidities | -0.79 | 0.009 | -0.74 | 0.008 | -0.89 | 0.003 | -0.65 | 0.033 |
| Advanced HIV | -4.99 | 0.001 | -4.71 | 0.001 | -4.69 | 0.059 | -4.78 | 0.001 |
| x Time (months) | 1.48 | 0.001 | 1.18 | 0.018 | 0.96 | 0.058 | 1.09 | 0.018 |
| x Time (months)^2^ | -0.11 | 0.012 | -0.09 | 0.067 | -0.07 | 0.161 | -0.09 | 0.049 |
| x Time (months)^3^ | 2.07e-3 | 0.053 | 2.06e-3 | 0.134 | 1.32e-3 | 0.256 | 2.12e-3 | 0.092 |
| Log(HIV RNA) | -0.54 | 0.011 | -0.41 | 0.039 | -0.35 | 0.059 | -0.40 | 0.062 |
| x Time (months) | 0.21 | 0.001 | 0.15 | 0.079 | 0.17 | 0.012 | 0.13 | 0.114 |
| x Time (months)^2^ | -0.02 | 0.003 | -0.01 | 0.153 | -0.02 | 0.033 | -0.01 | 0.166 |
| x Time (months)^3^ | 4.51e-4 | 0.005 | 3.06e-4 | 0.206 | 3.67e-4 | 0.059 | 2.91e-4 | 0.201 |
