# Supplementary material for: A practical evaluation of statistical methods for the analysis of patient reported outcomes in an observational pharmaceutical study
Source: PLoS One. 2026 Mar 18;21(3):e0344968. doi: 10.1371/journal.pone.0344968 (PMC12998841; doi:10.1371/journal.pone.0344968)
Supplement: Attachment — Submitted filename: Response to Reviewers.docx [file pone.0344968.s013.docx]

Dear PLOS One Editor and Reviewers,

Thank you for your review of the manuscript “A practical evaluation of statistical methods for the analysis of patient reported outcomes in an observational pharmaceutical study”. We appreciate the time you have taken on the review and the comments you provided.

Below we have provided responses to your comments.

Thank you for your time.

Best,

Heribert Ramroth

**----**

**Reviewer #1**

**General assessment**
The paper “A practical evaluation of statistical methods for the analysis of patient reported outcomes in an observational pharmaceutical study” is well-written, organized and statistically sound paper. It provides a comparative template of how longitudinal patient reported outcomes in HIV studies can be analyzed using four different statistical methods and which one should be chosen. However, the paper lacks novelty and reiterates what already exists in the literature. The authors have highlighted the assumptions and requirements of the methods, how these assumptions are violated or unmet in the PRO analyses, and which method can best handle them. But this is the very reason why the methods are being developed in the first place, and a study statistician analyzing the PRO data is expected to be aware of the assumptions and appropriateness of these methods in the study. Moreover, the pharmaceutical industry is well-aware of these methods and there appropriateness to their studies. Hence, this paper may be useful (if there is any) to a very limited audience working in the HIV studies with PROs.

**Response**: We thank reviewer one for the comment. We acknowledge that statisticians may be aware of the various methodologies, however, we disagree that this manuscript has a limited audience. The manuscript uses HIV data as an example of how to apply various methodologies for the analysis of PRO information. This does not limit the impact or applicability of the methodology to other therapeutic areas or to other industries (e.g., health care organizations, research institutions, etc.). The manuscript provides a methodologic comparison that will enable the wider audience to understand what direction would be appropriate for their analyses of PRO data.

Furthermore, the GEE approach is rarely used in the PRO field, despite its methodological advantages. We therefore would argue that the paper is novel and valuable in its illustration of the utility of the GEE, and that the code would be helpful for any statistician applying or adapting the method for a range of PRO data.

**----**

**Reviewer #2:**

**General assessment**
This manuscript addresses a highly relevant and timely topic in medical statistics and health outcomes research: the comparative evaluation of statistical methods for the analysis of patient-reported outcomes (PROs) in an observational HIV cohort. The authors present a practical application of four different methods (paired difference tests, Friedman’s ANOVA, linear mixed models – LMM, and weighted generalized estimating equations – wGEE) to assess longitudinal changes in SF-36 physical and mental health scores among treatment-naïve people living with HIV.
The paper is clearly written, and the methods are well motivated and generally appropriate for the stated aims. However, several important methodological and practical aspects of the statistical analysis require further clarification and strengthening, particularly in terms of assumptions, model diagnostics, missing data handling, and interpretability of results.

**Response**: Thank you for your comments. The commentary regarding further clarifications is presented below.

**----**

**Major Comment 1.** **Statistical assumptions and model validation**
While the manuscript appropriately highlights the violation of the MCAR assumption (via Little’s test) for the paired difference tests, there is no presentation of diagnostic checks for key assumptions underlying the LMM and GEE models.
Recommendation: Please include diagnostic plots or summaries to assess the normality of residuals, adequacy of random effects distribution, and the correlation structure used in the GEE framework.

**Response**: We thank reviewer two for their suggestion. We have added model diagnostic information of the linear mixed models to the Supplementary Figure S4*,* and referenced in the main text, line 231 “LMM model diagnostic plots are provided in Figure S4...”.

Please note that while there is some deviation at the tails of the distributions in the normality of residuals and random effects plots, the selected log transformation (PCSt = (–ln(100–PCS) and MCSt = (–ln(100–MCS)) was chosen because it satisfied the model assumptions most adequately from all transformations that were tested.

The only assumption of the generalised estimating equation (GEE) is that the working correlation matrix is correctly specified. We based our matrix selection on the model quasi-likelihood under the independence model criterion (QIC), with the lowest value representing the best model fit. This is a common approach for working correlation matrix selection [1]. As the unstructured matrix had the lowest QIC values, we selected this matrix for the MCS and PCS models. We have added a table of QIC values for each of the working correlation matrices to the Supplementary Materials: Supplementary Table 3, and referenced this in the main text, line 233: “The unstructured working correlation matrix was selected for all wGEE models (Table S4).”

**----**

**Major comment 2. Transformations and interpretability**
The log-transformation of the SF-36 outcomes for LMM (–ln(100 – score)) is statistically valid in the presence of ceiling effects but substantially reduces the interpretability of the resulting model coefficients.
Recommendation: Discuss this limitation more explicitly. Consider whether models such as GLMMs or quantile regression could provide more interpretable results without requiring transformation.

**Response**: We agree with Reviewer two that the log-transformation of the LMM is a major limitation of this statistical approach, as the model coefficients must be presented on this transformed scale. To improve the interpretability, a statistician may wish to generate back-transformed predictions for different patient profiles for the change in PRO over time (similar to the wGEE results presented in Figure 2). However, this may not be preferable if the model coefficients themselves are of interest, and in these cases, alternative approaches may be better, such as the (w)GEE, GLMMs or quantile regression.

We have added further elaboration on the limited interpretability of LMMs with a transformed outcome variable to the discussion, line 280: “The LMM analyses required transformation of the MCS and PCS outcomes to meet its normality assumptions, and due to its complexity, the parameter estimates couldn’t be back transformed so the interpretability of the model coefficients was reduced. This is a major limitation of LMMs when data do not satisfy its assumptions. In these cases, back-transforming model predictions and illustrating the change over time across patient profiles would support understanding”.

We have also noted where alternative methods to those illustrated in the manuscript to the discussion may be useful, line 331: “In this paper we have covered four statistical approaches, but depending on other researchers’ data, other methods may be considered. For example, when data cannot be assumed to satisfy the MCAR or MAR assumptions, pattern-mixture models or joint modelling approaches can better handle missing data. Data that conforms to the normal distribution assumption with or without a link function can be handled with the LMM or generalised LMM, giving greater interpretability that when using the LMM with a complex transformation of the outcome variable. A wider range of methods have been discussed theoretically elsewhere [1]. Future work would be valuable practically evaluating the applicability of these methods to PRO data.”
**----**

**Major comment 3. Model selection and covariate handling**
Covariates were selected using backward elimination based on p-values and AIC. Although common in practice, this approach can be unstable and data-dependent, especially in observational settings with potential confounding.
Recommendation: Consider validating the selected models via cross-validation or information-theoretic criteria across multiple imputations. Additionally, clarify how missing covariate data were handled.

**Response**: We appreciate the limitations of backwards selection and have now added a note on this to the methods, line 142: “As backwards selection can lead to inappropriate removal of important covariates, we then validated the covariate selection using the Akaike information criterion…”. Validating the models using cross-validation or multiple imputation would require more analysis time which we cannot allocate to this project. The AIC validation step only modified the covariate selection slightly, so we feel that the approach used is appropriate in this case.

Missing covariate data were handled using complete case analysis. 8 of 293 participants had missing covariate data, therefore we expect that this made little difference to the multivariable model results. We have noted this in the methods, lines 147 and 161: “Participants with missing covariate data were excluded from the LMM/wGEE analysis.”
**----**

**Major Comment 4. Weighted GEE models and missing data handling**
The use of inverse probability weighting to adjust for missingness is a strength of the paper. However, the logistic models used to compute weights are not reported in detail (e.g., predictor coefficients, diagnostics, AUC, Hosmer-Lemeshow tests).
Recommendation: Please provide summary tables or appendices reporting these models and their diagnostics. The reproducibility and credibility of the wGEE results depends on the robustness of this step.

**Response**: We thank reviewer two for their suggestion. We have added the model results and diagnostics to the Supplementary Table S3, and referenced this in the main text, line 231 “and the logistic regression results for the wGEE weighting models are presented in Table S3”. We have provided the results for the baseline (Month 0) model, which included the following predictor variables: HIV RNA (log_10_) at baseline, sex, age, number of neuropsychiatric comorbidities, number of physical comorbidities, and presentation with advanced HIV at baseline (yes/no). We have also provided the results for the Month 3, 6, 12, 18, and 24 models, which also included a variable for the most recently observed MCS and PCS score. Please note that for participants who did not have a value for the most recently observed MCS/PCS for a given follow-up visit window, we ran a separate model without these variables, and assigned the weight generated from this model for that participant’s visit. However, for simplicity, we have only presented the model results for this with the most recent MCS/PCS predictors. For each model, we performed the:

- Hosmer-Lemeshow test – p value was greater than 0.05 for all models, indicating no significant difference between observed and expected outcomes, and therefore that the logistic regression model fits the data well.
- Link function test - The second-order linear predictor was not statistically significant for all models, suggesting that the link function used (logit link) adequately captured the relationship between the predictors and the outcome.

We are therefore confident that the wGEE results are credible and reproducible.

**----**

**Major comment 5. Model comparison and time modeling**
The manuscript uses QIC to compare models with different time specifications. However, differences in QIC values are minimal, and the choice of the final model (e.g., fractional polynomial vs spline) is not strongly justified.
Recommendation: Discuss whether differences in model fit are statistically and practically meaningful. The use of simpler, more interpretable models (e.g., splines) may be preferable unless the polynomial forms provide substantial gains.

**Response**: We thank reviewer two for their comment. We recognise that there is little difference in QIC values across non-linear modelling approaches. We opted for the best-fitting model as this gave us a quantitative method of selection, compared basing our selection on interpretability, which would have been subjective. We agree that when other researchers are selecting models for their data, interpretability will be a priority and therefore simpler models may be more useful.

We have added some elaboration to our paragraph discussing this point in the discussion, line 300:

“The fractional polynomial and polynomial models gave the best fitting models for the MCS and PCS respectively. However, as both polynomial approaches use power terms to model non-linear trends, the parameter estimates themselves may be difficult to interpret for audiences without statistical training. In this illustration, we selected the best model based on model-fit, however for other researchers, the more interpretable piecewise linear spline approach may be preferable, particularly if the model fit is similar to (fractional) polynomial transformations. If selecting a polynomial or fractional polynomial approach, PRO trends can be visualised using the estimates generated from the model to aid communication of results. This visualisation could also be extended to generate subgroup-specific estimates and confidence intervals could be generated with bootstrapping. The decision on the best approach for other datasets will depend on the underlying trend being modelled.”
**----**

**Minor comment 1. Figures and tables:**

- Figure 2 could be enhanced with 95% confidence bands or bootstrapped intervals.
- Table 1 is informative but could include p-values comparing included vs excluded patients across methods to quantify potential bias due to differential inclusion.

**Response**: We thank the reviewer for their suggestion to improve figure 2, and we agree that 95% confidence intervals produced by bootstrapping would enhance the figure. However, due to time and resource constraints we are not able to generate these. We believe that the main messages of the figures are still communicated adequately, and we note that other statisticians applying these methods may choose to generate confidence intervals on predictions (line 307): “This visualisation could also be extended to generate subgroup-specific estimates and confidence intervals could be generated with bootstrapping”. We have added p-values to Table 1.
**----**

**Minor comment 2. Software and reproducibility:**
The authors refer to a GitHub repository, which is commendable. However, more specific references to scripts corresponding to each analysis would facilitate replication.

**Response**: The GitHub repository has each of the analyses clearly detailed in well-annotated, separate R markdown files (). We therefore believe that it is sufficient to simply link to the overall repository and allow the reader to select the analysis of interest.

**Minor comment 3. Sensitivity to MNAR:**
- The Authors briefly mention MNAR scenarios but only conduct sensitivity analyses under MAR assumptions. Approaches such as pattern-mixture models or joint modeling frameworks might provide deeper insight.
- Recommendation: If it is not feasible to implement, acknowledge this as a limitation and suggest directions for future work.https://github.com/lucyrose96/PRO-Methods-Sample-Code). We therefore believe that it is sufficient to simply link to the overall repository and allow the reader to select the analysis of interest.
**----**

**Minor comment 3. Sensitivity to MNAR:**
- The Authors briefly mention MNAR scenarios but only conduct sensitivity analyses under MAR assumptions. Approaches such as pattern-mixture models or joint modeling frameworks might provide deeper insight.
- Recommendation: If it is not feasible to implement, acknowledge this as a limitation and suggest directions for future work.

**Response**: As suggested by Reviewer 2, implementing these models would be time and resource intensive, and we believe beyond the scope of this manuscript. We have instead added a comment to the discussion, line 330, recognising that under scenarios where the missing not at random (MNAR) assumption is valid, other models would be more appropriate.

“In this paper we have covered four statistical approaches, but depending on other researchers’ data, other methods may be considered. For example, when data cannot be assumed to satisfy the MCAR or MAR assumptions, pattern-mixture models or joint modelling approaches can better handle missing data. Data that conforms to the normal distribution assumption with or without a link function can be handled with the LMM or generalised LMM, giving greater interpretability that when using the LMM with a complex transformation of the outcome variable. A wider range of methods have been discussed theoretically elsewhere [1]. Future work would be valuable practically evaluating the applicability of these methods to PRO data.”
**----**

**Conclusion**
This manuscript provides a valuable and practical comparison of statistical methods for the analysis of PROs in observational settings. The use of real-world data, a comprehensive set of methods, and sensitivity analyses is commendable. However, the paper would greatly benefit from:
- enhanced reporting of model diagnostics and assumptions,
- justification of transformation choices and model selection,
- more detailed presentation of the weighting mechanism in wGEE.

After addressing these points, the manuscript will make a meaningful contribution to the applied statistical literature on longitudinal PRO analysis in HIV care and beyond.

**Response**: We believe that our edits to the manuscript and supplementary materials now sufficiently address the comments made by Reviewer 2. We have provided further details on the model diagnostics and assumptions with text and supplementary tables and figures. We have elaborated on transformation choices and model selection. Finally, we have presented the results for the weighting mechanism of the wGEE.

**Reviewer references for Reviewer Comments**
1. Fitzmaurice GM, Laird NM, Ware JH. Applied Longitudinal Analysis. 2nd ed. Wiley; 2012.
2. Verbeke G, Molenberghs G. Linear Mixed Models for Longitudinal Data. Springer; 2000.
3. Salazar A et al. Simple GEEs and wGEEs in longitudinal studies with dropouts. Statistics in Medicine. 2016;35:3424–3448.
4. Carpenter JR, Kenward MG. Multiple Imputation and Its Application. Wiley; 2013.
5. Royston P, Altman DG. Regression using fractional polynomials. Applied Statistics. 1994;43:429–467.

**Author references**

1. Wang YG, Fu L. Selection of working correlation structure in generalized estimating equations. Statistics in medicine. 2017 Jun 30;36(14):2206-19.
