# Supplementary material for: A practical evaluation of statistical methods for the analysis of patient reported outcomes in an observational pharmaceutical study
Source: PLoS One. 2026 Mar 18;21(3):e0344968. doi: 10.1371/journal.pone.0344968 (PMC12998841; doi:10.1371/journal.pone.0344968)
Supplement: Attachment — Submitted filename: REVIEW PONE-D-24-44219R1.docx [file pone.0344968.s012.docx]

Thank you for the opportunity to review this interesting and well-prepared manuscript. The study provides a clear and structured comparison of statistical methods commonly used for analyzing longitudinal patient-reported outcomes (PROs) in an observational cohort of HIV-infected patients. The manuscript is technically sound, the methods are rigorously implemented, and the results support the conclusions drawn. The writing is clear and of high quality.

Below, I provide comments aimed at enhancing the conceptual clarity and interpretive transparency of the manuscript.

Key Comments

Clarify the implicit baseline model used to evaluate the performance of statistical methods.

Throughout the manuscript, method comparisons are partly interpreted in relation to an expected clinical trajectory (e.g., an initial improvement in MCS/PCS followed by a plateau). While this expectation is consistent with previous literature, it effectively serves as an implicit baseline for assessing method appropriateness. Because the true underlying trajectory is unknown, it would be helpful to explicitly acknowledge that method performance is evaluated in terms of consistency with clinically plausible trends, rather than accuracy relative to a known ground truth.

Lack of an objective performance benchmark.

Because the analysis relies exclusively on real observational data, it is inherently descriptive. While appropriate for the stated objectives, the lack of simulation-based analyses limits the ability to quantify the bias or robustness of each method in controlled missingness scenarios. Even a brief simulation (e.g., in the Supplementary Material) using known missingness trajectories and mechanisms would substantially strengthen the methodological conclusions.

Contextualization of the selected methods.

The manuscript focuses on four approaches commonly used in practice, which is reasonable. However, a brief explanation of why other families of longitudinal models (e.g., nonlinear mixed models, joint models, or survival-type "time-to-plateau" approaches) were not considered would help define the scope of the work and guide future extensions.

Interpretation of LMM Transformations.

The need to log-transform the results for LMM normality assumptions reduces interpretability. While this limitation is acknowledged, I suggest highlighting it more explicitly as part of the trade-off between model assumptions and interpretability, especially since one of the objectives of the manuscript is to assess the communicability of the results.

Minor Comments

Figure 2 could benefit from scaling the axes to better highlight the initial sharp increase in MCS.

Ensure consistent terminology for physical health-related quality of life versus PCS throughout the text.

Please clarify in the Methods section whether "time" has been coded in months as numeric or categorical for continuous-time wGEE analyses.

Where both AIC and QIC are referenced, ensure the rationale for their use is clearly stated.

Data Availability

Although the ethical constraints regarding patient data are understandable, the manuscript does not fully meet PLOS ONE data availability requirements. Only the sample R code is publicly available, and the underlying dataset cannot be shared. It may be helpful to provide more detailed guidance to researchers on how to access restricted data (e.g., through a controlled application process), if applicable.

Conclusion

Overall, this is a well-executed and valid applied methodological study. After addressing the above clarifications, the manuscript will be further strengthened and will provide clearer guidance for practitioners working with PRO data in observational settings. I recommend acceptance after minor revisions.
