# Supplementary material for: A practical evaluation of statistical methods for the analysis of patient reported outcomes in an observational pharmaceutical study
Source: PLoS One. 2026 Mar 18;21(3):e0344968. doi: 10.1371/journal.pone.0344968 (PMC12998841; doi:10.1371/journal.pone.0344968)
Supplement: Attachment — Submitted filename: Response_to_Reviewers_auresp_2.docx [file pone.0344968.s014.docx]

Dear PLOS One Editor and Reviewers,

Thank you for your review of the manuscript “A practical evaluation of statistical methods for the analysis of patient reported outcomes in an observational pharmaceutical study”. We appreciate the time you have taken on the review and the comments you provided.

Below we have provided responses to your comments.

Thank you for your time.

Best,

Heribert Ramroth

__________________________________________________________________

**Reviewer’s comments:**

Thank you for the opportunity to review this interesting and well-prepared manuscript. The study provides a clear and structured comparison of statistical methods commonly used for analyzing longitudinal patient-reported outcomes (PROs) in an observational cohort of HIV-infected patients. The manuscript is technically sound, the methods are rigorously implemented, and the results support the conclusions drawn. The writing is clear and of high quality.

Below, I provide comments aimed at enhancing the conceptual clarity and interpretive transparency of the manuscript.

**Key Comments**

1. **Clarify the implicit baseline model used to evaluate the performance of statistical methods.**

Throughout the manuscript, method comparisons are partly interpreted in relation to an expected clinical trajectory (e.g., an initial improvement in MCS/PCS followed by a plateau). While this expectation is consistent with previous literature, it effectively serves as an implicit baseline for assessing method appropriateness. Because the true underlying trajectory is unknown, it would be helpful to explicitly acknowledge that method performance is evaluated in terms of consistency with clinically plausible trends, rather than accuracy relative to a known ground truth.

**Response**: We thank the reviewer for their helpful observation. We agree that our comparisons implicitly relied on clinically plausible expectations of change (e.g., initial improvement in MCS/PCS followed by a plateau), as suggested by previous literature. To clarify this, we have revised the manuscript to explicitly state that method performance was evaluated in terms of consistency with these expected trends rather than accuracy against a known ground truth, since the true underlying trajectory is unknown in observational HIV studies. The revised text is in the Methods, line 103: “*As the true underlying change in MCS and PCS is unknown, we compare methods on their consistency with clinically plausible trends*”, and the Discussion, line 308: “*As we used observational data, it is not possible to know if this trend represents the true underlying MCS and PCS trends, however it corroborates previously seen trends in physical and mental HRQoL following initiation of antiretrovirals in treatment-naïve PLWH*”.

2. **Lack of an objective performance benchmark.**

Because the analysis relies exclusively on real observational data, it is inherently descriptive. While appropriate for the stated objectives, the lack of simulation-based analyses limits the ability to quantify the bias or robustness of each method in controlled missingness scenarios. Even a brief simulation (e.g., in the Supplementary Material) using known missingness trajectories and mechanisms would substantially strengthen the methodological conclusions.

**Response**: We agree with the reviewer that in order to quantify the degree of bias and accuracy of each statistical approach, a simulation study would be desirable. To do this properly, a range of plausible scenarios should be simulated to test the sensitivity of the results to the scenario that is given. It would require significant time and resource to test the alternative MCS/PCS trajectories, intermittent and dropout missingness, skewed data distributions, etc. We believe that this would also be a valuable contribution to the literature but is ultimately enough content for a separate manuscript. For our manuscript, the primary objective was to evaluate statistical approaches on data that reflect the complexity and variability of real-world clinical practice.

We have therefore instead added some text to the Discussion, line 337, to recommend simulations in future studies: “*While the primary objective of this manuscript was to evaluate statistical approaches on data that reflect the complexity and variability of real-world clinical practice, simulations would be valuable to quantify the degree of accuracy and precision of each method. A simulation study was beyond the scope of this manuscript, but future work evaluating statistical methods on simulated PRO data would be valuable.*”

3. **Contextualization of the selected methods.**

The manuscript focuses on four approaches commonly used in practice, which is reasonable. However, a brief explanation of why other families of longitudinal models (e.g., nonlinear mixed models, joint models, or survival-type "time-to-plateau" approaches) were not considered would help define the scope of the work and guide future extensions.

**Response**: We selected methods that are either commonly used (paired difference test, ANOVA) or have been proposed for PRO analyses but not frequently taken up (LMM, GEE). We have added this contextualisation when introducing the selected approaches in the Methods, line 112: *“We selected methods that are either commonly used (PD-test, F-ANOVA) or have been proposed for PRO analyses but not frequently taken up (LMM, GEE) [1,26,39].*”. We also previously had a paragraph discussing alternative approaches that may be favourable for other datasets. We have elaborated on this paragraph with the methods suggested by this reviewer. Discussion, line 348:

*“In this paper we have covered four statistical approaches, but depending on other researchers’ data, other methods may be considered. For example, when data cannot be assumed to satisfy the MCAR or MAR assumptions, pattern-mixture models or joint modelling approaches can better handle missing data. Data that conforms to the normal distribution assumption with or without a link function can be handled with the LMM or generalised LMM, giving greater interpretability that when using the LMM with a complex transformation of the outcome variable. PROs may also be analysed as time to plateau or time to deterioration, in which case, survival-type approaches would be more appropriate than those covered in this manuscript. A wider range of methods have been discussed theoretically elsewhere [1], and future work would be valuable practically evaluating the applicability of these methods to PRO data.”*

4. **Interpretation of LMM Transformations.**

The need to log-transform the results for LMM normality assumptions reduces interpretability. While this limitation is acknowledged, I suggest highlighting it more explicitly as part of the trade-off between model assumptions and interpretability, especially since one of the objectives of the manuscript is to assess the communicability of the results.

**Response**: We thank the reviewer for their suggestion. We would like to highlight that we have addressed this in detail in the discussion, line 287:

*“The LMM analyses required transformation of the MCS and PCS outcomes to meet its normality assumptions, and due to its complexity, the parameter estimates couldn’t be back transformed so the interpretability of the model coefficients was reduced. This is a major limitation of LMMs when data do not satisfy its assumptions. In these cases, back-transforming model predictions and illustrating the change over time across patient profiles would support understanding. The issue with LMM interpretability contrasts to the more robust and flexible wGEE approach, which doesn’t make distributional assumptions, therefore facilitating analysis on the original scale”*

However, we appreciate the suggestion to frame it within the overarching statistical appropriateness vs interpretability trade-off, so have added some further discussion in an additional paragraph, line 297:

“The methods evaluated in this manuscript highlight an inherent trade-off in study design between model complexity/appropriateness of assumptions, and interpretability. Typically, simpler methods are more easily communicated, particularly to audiences without statistical training. More complex models may more accurately represent the data but the underlying message can be lost. Ultimately, method selection should strike an appropriate balance for the study objectives.”

**Minor Comments**

5. **Figure 2 could benefit from scaling the axes to better highlight the initial sharp increase in MCS.**

**Response**: We have reviewed Figure 2 and believe that the scaling of the axes sufficiently represents the MCS and PCS trajectories. The scales chosen are by intention the same on each y axis (increments of 2.5), but start from a different value. The initial increase in MCS is apparent and more clearly communicated with the numerical annotations.

6. **Ensure consistent terminology for physical health-related quality of life versus PCS throughout the text.**

**Response**: We have now ensured that we exclusively refer to our results as MCS and PCS. As these are representative of metal and physical health-related quality of life, we also refer to these terms in the discussion.

7. **Please clarify in the Methods section whether "time" has been coded in months as numeric or categorical for continuous-time wGEE analyses.**

**Response**: We have added some clarification to the methods to highlight the use of a time variable in numeric months, line 168: “*To compare alternative non-linear modelling approaches for PRO analyses, the wGEE analysis was repeated with time as a continuous, numeric, non-linear variable”*

8. **Where both AIC and QIC are referenced, ensure the rationale for their use is clearly stated.**

**Response**: We thank the reviewer for picking up on readers’ likely unfamiliarity with the QIC. We assume that readers are likely to have more understanding of commonly used AIC, so we have left the introduction to that as it is: line 146 “we then validated the covariate selection using the Akaike information criterion (AIC) and including only those that improved the model fit (lower AIC)”. However, we have added further explanation to the introduction of the QIC, line 164: *“The QIC was developed as a modification of the AIC to apply to models fit by the GEE approach. Therefore, in this manuscript we use the QIC for GEE analyses and AIC for LMM analyses.”*

9. **Data Availability**

Although the ethical constraints regarding patient data are understandable, the manuscript does not fully meet PLOS ONE data availability requirements. Only the sample R code is publicly available, and the underlying dataset cannot be shared. It may be helpful to provide more detailed guidance to researchers on how to access restricted data (e.g., through a controlled application process), if applicable.

**Response**: We have consulted with the Gilead Sciences Data Sharing team and have added a company-approved statement on data availability to the end of the manuscript, line 378:

*“Gilead Sciences shares anonymised individual patient data upon request or as required by law or regulation with qualified external researchers based on submitted curriculum vitae and reflecting non conflict of interest. The request proposal must also include a statistician. Approval of such requests is at Gilead Science’s discretion and is dependent on the nature of the request, the merit of the research proposed, the availability of the data, and the intended use of the data. Data requests should be sent to* [*datasharing@gilead.com*](mailto:datasharing@gilead.com)*.”*

**Conclusion**

Overall, this is a well-executed and valid applied methodological study. After addressing the above clarifications, the manuscript will be further strengthened and will provide clearer guidance for practitioners working with PRO data in observational settings. I recommend acceptance after minor revisions.

**Response**: We thank this reviewer for their comments and suggestions. We believe that the manuscript is now improved and agree that it will be a valuable tool for researchers utilising observational PRO data.

**References**

**1.** Coens C, Pe M, Dueck AC, Sloan J, Basch E, Calvert M, et al. International standards for the analysis of quality-of-life and patient-reported outcome endpoints in cancer randomised controlled trials: recommendations of the SISAQOL Consortium. Vol. 21, The Lancet Oncology. 2020. p. e83–96.

**26.** Bell ML, Horton NJ, Dhillon HM, Bray VJ, Vardy J. Using generalized estimating equations and extensions in randomized trials with missing longitudinal patient reported outcome data. Psychooncology. 2018;27(9):2125–31.

**39.** Hamel JF, Saulnier P, Pe M, Zikos E, Musoro J, Coens C, et al. A systematic review of the quality of statistical methods employed for analysing quality of life data in cancer randomised controlled trials. Vol. 83, European Journal of Cancer. 2017. p. 166–76.
